# Supplementary material for: MLR and dMLR Predict Locoregional Control and Progression-Free Survival in Unresectable NSCLC Stage III Patients: Results from the Austrian Radio-Oncological Lung Cancer Study Association Registry (ALLSTAR)
Source: J Clin Med. 2025 Dec 15;14(24):8876. doi: 10.3390/jcm14248876 (PMC12734154; doi:10.3390/jcm14248876)
Supplement: Supplementary file 1 [file jcm-14-08876-s001.zip › jcm-3962885-supplementary.pdf]

Supplementary Table S1. Summary of all the tested biomarkers

		Biomarker					
		N = 183			N =112		
		Threshold interval (p<0.05)	Threshold	P-value for threshold	Threshold interval (p<0.05)	Threshold	P-value for threshold
NLR	LRC	none	none	none	none	none	none
	PFS	2.842*	2 842	0.043	12.75-13.27	12.75	0.029
	OS	2.895-2.906	2 906	0.031	24.088*	24 088	0.017
MLR	LRC	0.663-0.696	0.665	0.018	0.663-0696	0.665	0.030
	PFS	0.450-0.786	0.665	<0.001	0.420 - 0.677	0.665	0.004
	OS	none	none	none	none	none	none
PLR	LRC	79.091*	79 091	0.013	none	none	none
	PFS	71.777*	71 777	0.050	79.091*	79.091	0.046
	OS	none	none	none	79.091 or 597.895**	79.091	0,009
dNLR	LRC	3.876-4.293	4	0.037	none	none	none
	PFS	none	none	none	none	none	none
	OS	4.255-4.364	4.293	0.025	3.815-3.876	3.815	0.030
LDH	LRC	152-155	154	0.012	146 - 153	153	0.010
	PFS	108*	108	0.042	177*	177	0.037
	OS	324-331	327	<0.001	194*	194	0.039
dMLR	LRC	0.945-0.987	0.945	0.054	1.173*	0.945	0.209
	PFS	0.58-1.077	0.945	<0.001	0.769-1,033	0.945	0.003
	OS	0.58-0.688	0.6	0.015	none	none	none

Supplementary figures

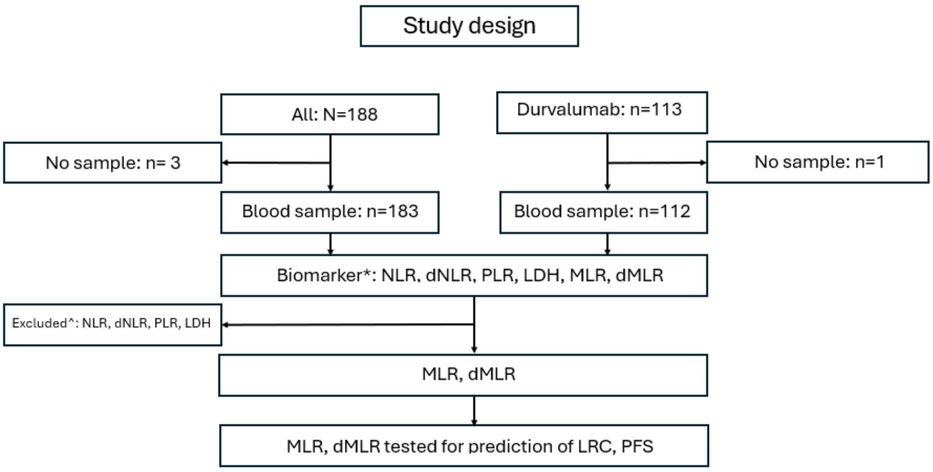

Supplementary Figure S1.
*Biomarkers that were previously tested in another RWD study (Park PACIFIC-KR, reference [13])
^These biomarkers did not fulfill the inclusion criteria for further investigation defined in methods section 2.4.

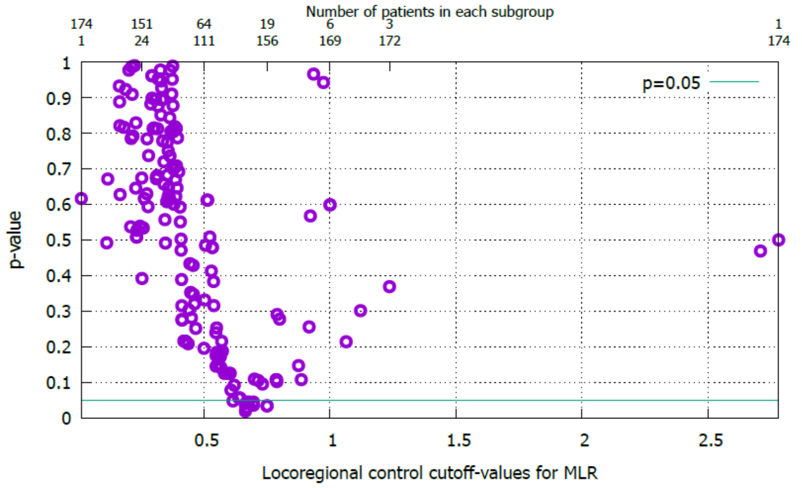

Supplementary Figure S2. Locoregional control: MLR cutoff values.

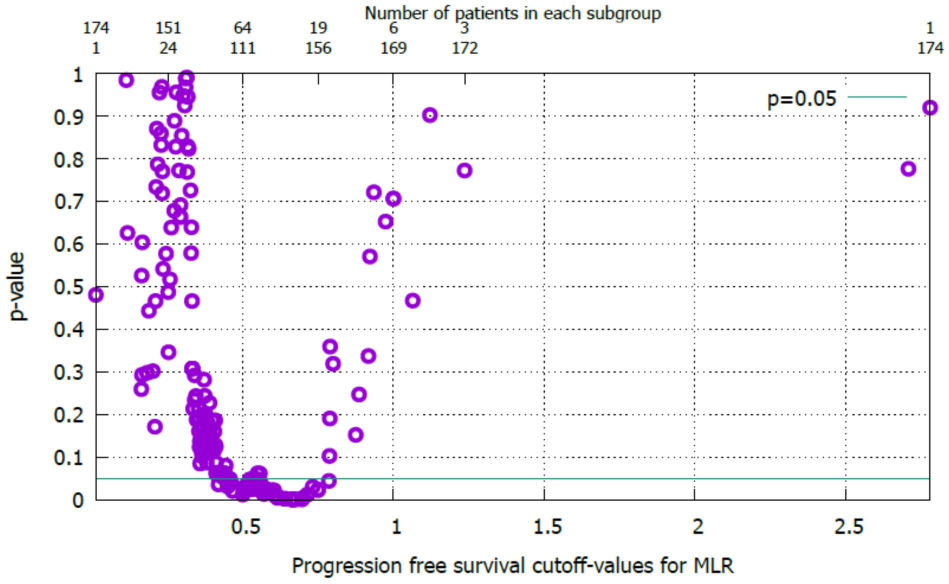

Supplementary Figure S3. Progression free survival: MLR cutoff values.

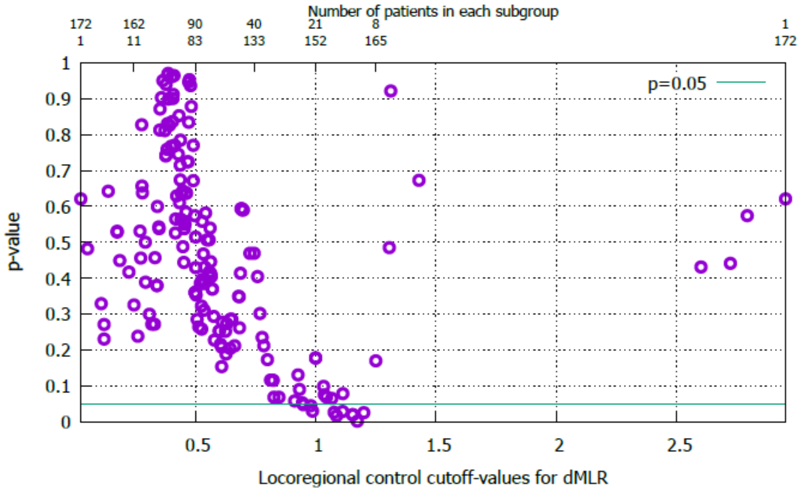

Supplementary Figure S4. Locoregional control: dMLR cutoff values.

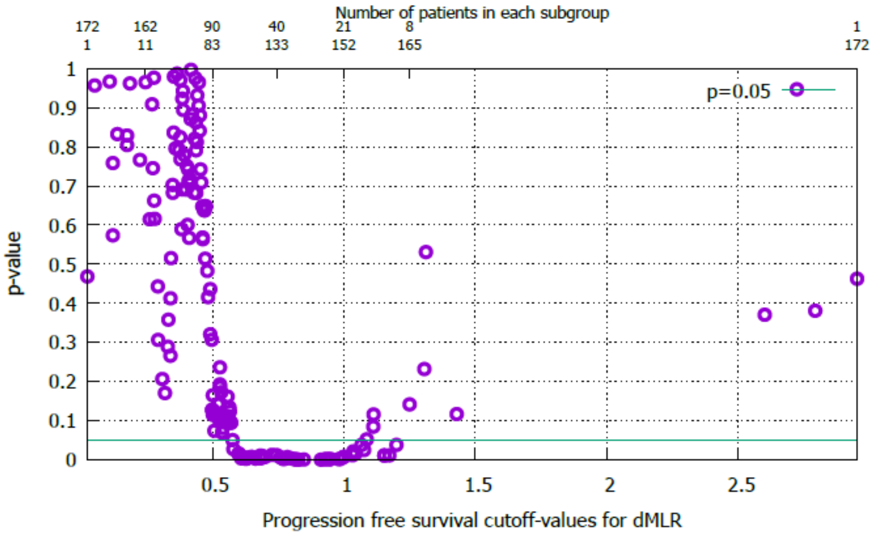

Supplementayr Figure S5. Progression free survival: dMLR cutoff values.
